# Safety and Feasibility of Antibiotic De-escalation in Critically Ill Children With Sepsis – A Prospective Analytical Study From a Pediatric ICU

**DOI:** 10.3389/fped.2021.640857

**Published:** 2021-03-08

**Authors:** Vasudha Battula, Ravi Kumar Krupanandan, P. Senthur Nambi, Bala Ramachandran

**Affiliations:** ^1^Department of Pediatric Critical Care, Kanchi Kamakoti CHILDS Trust Hospital, Chennai, India; ^2^Department of Pediatric Infectious Diseases, Kanchi Kamakoti CHILDS Trust Hospital and The CHILDS Trust Medical Research Foundation, Chennai, India

**Keywords:** antibiotic de-escalation, antimicrobial resistance, culture negative sepsis, critically ill children, de-escalation in ICU

## Abstract

**Introduction:** De-escalation is the key to balance judicious antibiotic usage for life-threatening infections and reducing the emergence of antibiotic resistance caused by antibiotic overuse. Robust evidence is lacking regarding the safety of antibiotic de-escalation in culture negative sepsis.

**Materials and Methods:** Children admitted to the PICU during the first 6 months of 2019 with suspected infection were included. Based on the clinical condition, cultures and septic markers, antibiotics were de-escalated or continued at 48–72 h. Outcome data like worsening of primary infection, acquisition of hospital acquired infection, level of ICU support and mortality were captured.

**Results:** Among the 360 admissions, 247 (68.6%) children received antibiotics. After excluding 92 children, 155 children with 162 episodes of sepsis were included in the study. Thirty four episodes were not eligible for de-escalation. Among the eligible group of 128 episodes, antibiotics were de-escalated in 95 (74.2%) and continued in 33 (25.8%). The primary infection worsened in 5 (5.2%) children in the de-escalation group and in 1 (3%) in non de-escalation group [Hazard ratio: 2.12 (95%CI: 0.39–11.46)]. There were no significant differences in rates of hospital acquired infection, mortality or length of ICU stay amongst the groups. Blood cultures and assessment of clinical recovery played a major role in de-escalation of antibiotics and the clinician's hesitation to de-escalate in critically ill culture negative children was the main reason for not de-escalating among eligible children.

**Conclusion:** Antibiotic de-escalation appears to be a safe strategy to apply in criticallly ill children, even in those with negative cultures.

## Introduction

The SPROUT study on global epidemiology of pediatric sepsis has shown the prevalence of sepsis to be 8.2% among ICU admissions and the hospital mortality in septic children was 25% (1). Various other studies have shown pediatric sepsis mortality varying from 10 to 60% (1–3). Surviving Sepsis Guidelines advocate administration of antibiotics as soon as possible, within 1 h to children with septic shock and within 3 h to children with sepsis-associated organ dysfunction without shock (4). It is quite challenging to confirm infection within such short time spans, especially in younger children with numerous infection mimics. Even in case of infection, it is not possible to identify the specific causative agent immediately. In this context, it is imperative to administer empiric broad spectrum antibiotics early in a critically ill septic child in the emergency room or PICU soon after presentation.

Antibiotics have saved millions of lives since their discovery. Due to injudicious overuse and misuse, microbes have developed resistance to antibiotics (5). Studies have shown significant correlation between carbapenem usage and the prevalence of carbapenem resistance across Intensive Care Units (6). Almost all the antibiotics discovered so far are endangered by resistance (7). The rapidity at which antimicrobial resistance is emerging surpasses the pace at which new antimicrobials are developed, placing us in an “antimicrobial resistance crisis.”

Antimicrobial stewardship emerged as a potential solution for the problem of antimicrobial resistance. One of the key strategies of antibiotic stewardship guidelines is antibiotic de-escalation (8). Though there is no available consensual definition, de-escalation is accepted as reviewing empirical broad spectrum antibiotics after 48–72 h after initiation, with available microbiological reports and the patient's clinical condition and either stopping the antibiotic or changing to a narrow spectrum drug or decreasing the number of antibiotics (9). De-escalation not only prevents the emergence of antimicrobial resistance, but also protects from adverse effects of broad spectrum antibiotics and brings down the cost of anti-microbial therapy. Though de-escalation is advocated as a crucial strategy of antimicrobial stewardship, it is not universally practiced. Studies have shown conflicting results regarding the safety of de-escalation (10, 11). The MERINO trial has shown that mortality rates are lower with the use of carbapenems, rather than Beta-lactam beta-lactamase inhibitor (BL-BLI) combination for infections caused by Extended Spectrum Beta Lactamase producing bacteria, though the clinical and bacteriological resolution of infections was not different between the two groups (12).

In a microbiologically confirmed infection, it is easy to decide on changing antibiotics based on susceptibility reports. But in case of children with clinically suspected infection, without microbiological confirmation or definite alternate diagnosis, it is not easy to rule out bacterial infection and de-escalate. Children receiving antibiotics in primary centers before referral, those with pneumonia and certain immune deficiencies will have low bacteriological yields on blood cultures. In such scenarios, we have to consider multiple factors other than microbiological reports, to decide on safe de-escalation. There are no studies focusing on safety of antibiotic de-escalation in critically ill children. In order to address the lacunae in available evidence regarding the safety of antibiotic de-escalation and to understand the factors effecting the decision of de-escalation, we analyzed our de-escalation practice and its safety for a period of 6 months in our PICU.

## Materials and Methods

This was a prospective analytical observational study conducted between January and June 2019 in the 14 bedded multidisciplinary PICU at Kanchi Kamakoti CHILDS Trust Hospital, a 200-bed tertiary care children's hospital in Chennai, India. The study was approved by the hospital Institutional Review Board. Ethical committee has waived the participation consent as the study was observational and was not involving any intervention. Our PICU is a closed ICU managed by Paediatric Intensivists. Children from 1 month to 18 years of age admitted to the PICU for sepsis or developing sepsis after admission and receiving empiric antibiotics were included. As per the existing pediatric sepsis definition, sepsis was considered as systemic inflammation (Systemic Inflammatory Response Syndrome) with presumed or proven infection (4, 13). Post-operative admissions and those who died or were transferred from the PICU within 48 h, before the availability of culture reports, were excluded. For all children with suspected sepsis, empiric antibiotics were started after sending two separate aerobic blood and other appropriate cultures (Urine/Endotracheal aspirate/Pus). Blood culture sampling volume in our PICU follows a standardized weight based protocol. Each blood culture set consists of two aerobic cultures drawn from different sites, with a blood volume between 2 and 20 ml each depending on the weight of the child. In children with indwelling central venous catheters, one culture is drawn through the catheter and the other from a peripheral vein. Empirical antibiotic therapy was chosen based on the common expected pathogen for the age of the child, site and severity of infection, immune status of the child and hospital flora (for Hospital acquired infection). Along with cultures, other tests like serological testing for appropriate infectious etiology (antigen/antibody testing for dengue fever, scrub typhus, malaria), Genexpert for tuberculosis, Multiplex film-array PCR of respiratory secretions in pneumonia, CSF in meningitis, Chest X-rays and other body imaging studies, total WBC counts, differential counts and C-Reactive protein in young infants were performed wherever indicated. Procalcitonin was measured in very few cases. After 48–72 h, children were reviewed clinically, with available culture and other lab reports. Though most cultures show growth signal in <48 h, time duration of 72 h was chosen for allowing enough time for species identification and sensitivity. The Protocol for the change of antibiotic is as follows: Antibiotics were changed as per culture reports in microbiologically confirmed sepsis. Antibiotics were stopped in children with alternate infectious or non-infectious etiology and de-escalated in children with culture negative sepsis (48 h), if they were improving clinically. Clinical improvement was considered as resolution or improvement of the symptoms and signs of sepsis. Hemodynamic stability and underlying immunodeficiency were taken into account. When antibiotics were stopped or changed to a narrower spectrum or reduced in number, it was considered as de-escalation. Changing the route of administration or duration of antibiotic course was not considered as de-escalation. The decision about antibiotics was taken by the ICU Consultant after discussing with the ICU team. Whenever the ICU Consultant is unable to make the decision or there is disagreement among the team the Infectious diseases team was involved. The antibiotic de-escalation was going on in the unit long before the initiation of the study. Not all intensivists have the same level of motivation to de-escalate. These practice variations gave us the opportunity to audit de-escalation and to look at the outcomes of de-escalated and non de-escalated groups.

Data relevant to ICU admission, indication for antibiotics, prior and current antibiotic therapy, clinical status, culture reports, biomarkers of sepsis, change of antibiotic, improving/worsening sepsis, hospital acquired infection after changing antibiotics, ICU supports required, length of ICU stay and hospital mortality were recorded by a PICU fellow after daily rounds. The reasons for changing or continuing the antibiotics were also recorded to look at the feasibility of de-escalation.

Mortality is multifactorial in critically ill children whereas clinical deterioration is the direct adverse effect that can happen due to de-escalation. Therefore, we have looked at the clinical deterioration after the antibiotic de-escalation as our primary outcome. “Clinical deterioration” was defined as clinical worsening of the symptoms like fever, respiratory distress, hemodynamics etc., or the signs of sepsis requiring escalation of antibiotics within 3 days after de-escalation. In children with positive culture before de-escalation, repeat culture has to grow the same organism in order to attribute clinical deterioration to de-escalation. In children with negative cultures, no other cause should be identified for this new worsening. Hospital acquired infections (HAI), length of the ICU stay, hospital mortality and sepsis attributable mortality were the secondary outcomes.

### Statistics

Quantitative variables were expressed as median with inter-quartile range and categorical variables were expressed as frequency with percentage. Mann Whitney *U*-test was used for comparing continuous variables and categorical variables were compared using Chi square or Fisher test, as appropriate. Primary and secondary outcome data were analyzed using time to event analysis, hazard ratio. While calculating hazard ratios, Log rank test was used to calculate chi square statistics, the *P*-value, and the confidence intervals. The time of de-escalation was taken as time zero. Other categorical variables, where time of occurrence was not of concern were analyzed using uncorrected Chi-square test or Fisher's exact test. Confidence level was taken as 95%. For all statistical analysis *P*-value <0.05 was considered statistically significant. For positive association between outcome and exposure, hazard ratio or odds ratio of more than one with confidence limits above one was taken.

## Results

**Figure 1:**
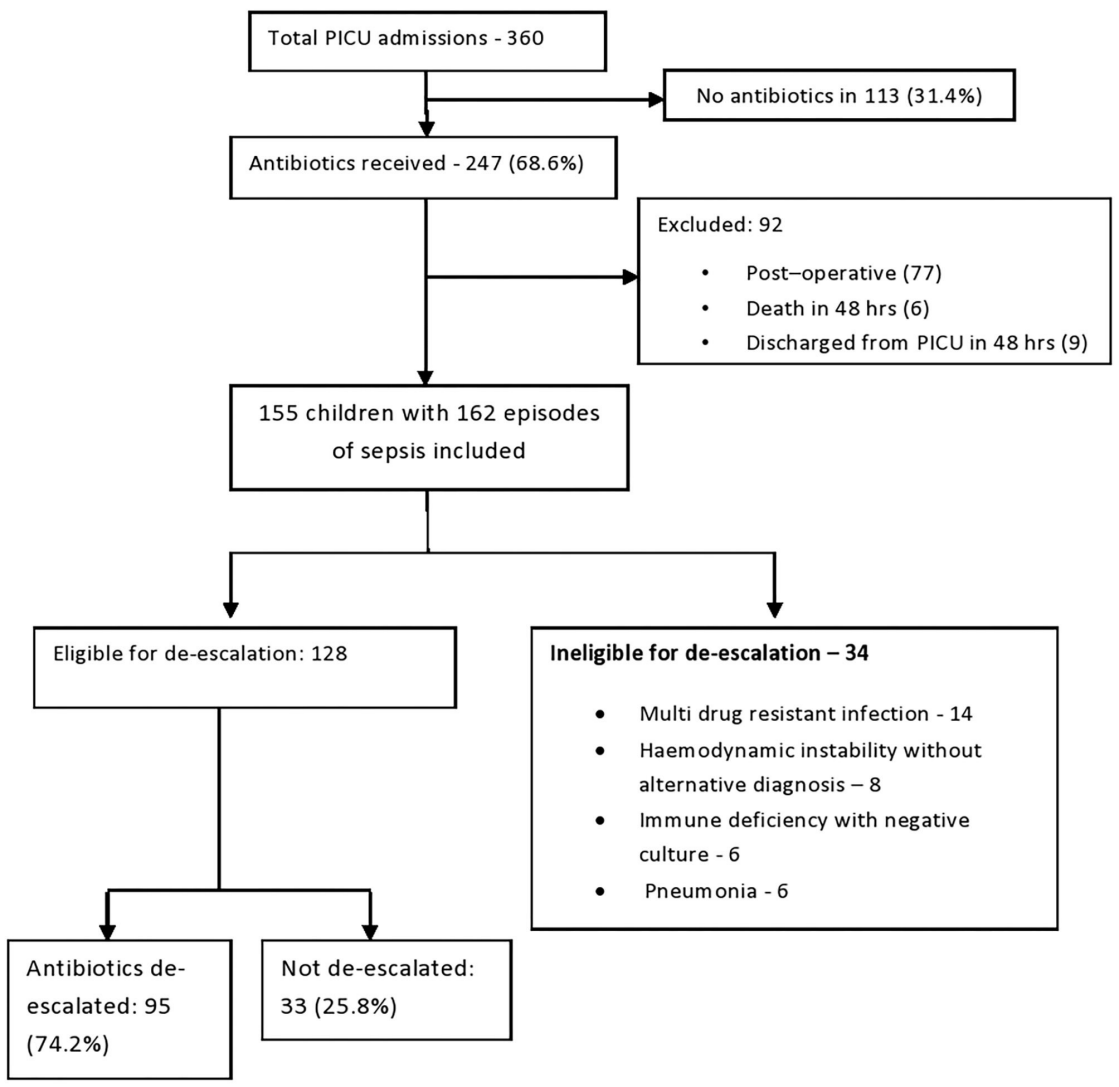
Patient flow diagram.

There were 360 PICU admissions during the study period, out of which, 247 (68.6%) received antibiotics. Children admitted for post operative care (*n* = 77) and children who died (*n* = 6) or were discharged (*n* = 9) from PICU within 48 h were excluded. The remaining 155 children with 162 episodes of sepsis were included in the study (Figure 1). Prior to arrival to our ICU, 31.4% of children has received antibiotics at the referral center or in the ward. Among the 162 episodes, 152 were primary infections and 10 were HAI. All HAIs were either blood stream or burn wound infections (Table 1). Empiric antibiotic combinations were used in 43.8%. Third generation Cephalosporins were used in 32%, BL-BLI combinations in 36% and Carbapenems in 22.2%. Vancomycin was a part of combination in 25.9% of the episodes.

**Table 1:** Patient demographics.

	**De-escalation group (*n* = 95)**	**Non de-escalation group (*n* = 33)**	***P*-Value**
Median age in months (inter quartile range)	40 (16–96)	32 (12–96)	0.09
Sex: Male, *n* (%)	53 (55.7)	20 (60.6)	0.63
Immune compromised, *n* (%)	11 (11.5)	3 (9)	0.98
MODS, *n* (%)	15 (15.7)	3 (9)	0.52
Shock at admission, *n* (%)	24 (25.2)	6 (18.1)	0.41
**Suspected site of infection**, ***n*** **(%)**
Blood	53 (55.7)	18 (54.5)	0.90
Respiratory system	23 (24.)	7 (21.2)	0.73
Central nervous system	21 (22.1)	6 (18.1)	0.63
Urinary tract infection	2 (2.1)	0 (0)	>0.99
Skin and soft tissue	7 (7.3)	2 (6.1)	0.85
Abdomen	2 (2.1)	0 (0)	>0.99
**ICU support**, ***n*** **(%)**
Ventilation	30 (31.5)	13 (39.3)	0.41
Inotropes	18 (18.9)	6 (18.1)	0.92
RRT	7 (7.3)	1 (3)	0.68
Culture positivity, *n* (%)	23 (24.2)	3 (9)	0.06
Alternate infectious etiology, *n* (%)	9 (9.4)	8 (24.2)	0.03
Alternate non –infectious etiology, *n* (%)	9 (9.4)	1 (3)	0.23

*MODS, Multi organ dysfunction syndrome; RRT, renal replacement therapy*.

After 48–72 h, de-escalation of antibiotics was considered. Cultures identified a pathogen in 37 of 162 (22.8%) episodes of suspected sepsis. Among positive cultures, 66% grew Gram negative bacilli, 32% Gram positive cocci and 2% fungi. Thirty six percentage of the isolates were multidrug resistant (Table 2). An alternate diagnosis was available in 27 (16.6 %) episodes by 72 h. Underlying immune-deficiency was present in 25 children (15.4%). The Infectious disease team was involved in 12% of cases.

**Table 2:** Profile of organisms isolated from cultures in the study.

**Organism**	**Total**	**Multi drug resistant**	**Extensive drug resistant**	**Pan drug resistant**
Gram Positive organisms	13	4	0	0
Enterobacteriaceae	20	10	4	0
Non enterobacteriaceae	7	5	2	0

Out of the 162 episodes of sepsis included in the study, 34 (20.9%) were ineligible for de-escalation. This group included: (1) Children with cultures growing multidrug resistant infections requiring continuation of broad spectrum antibiotics or escalation (*n* = 14), (2) Haemodynamically unstable children with negative cultures without alternative diagnosis after adequate evaluation (*n* = 8), (3) Immunodeficient children without clinical improvement, with negative cultures and without alternative diagnosis (*n* = 6) and (4) Non-ventilated children with features favoring bacterial pneumonia with negative blood cultures and negative respiratory viral PCR (*n* = 6). Lower respiratory secretions cannot be cultured in young children with pneumonia, without invasive ventilation or broncho-alveolar lavage and blood culture yield in severe pneumonia is <10% (14). After excluding ineligible children, 128 (79%) were eligible for de-escalation and were analyzed. In 128 episodes, 23 had positive cultures and 105 were culture negative and all these children were considered eligible for antibiotic de-escalation. Among them 95 (74%) underwent de-escalation and 33 (26%) did not undergo de-escalation. The primary outcome, clinical deterioration happened in 5 (5.2%) children after de-escalation and in one child (3%) continued on empirical antibiotics [Hazard ratio: 1.76 (95%CI: 0.28–10.91) *P*-value: 0.591]. De-escalation group had 8 (8%) and non de-escalation group had 6 (18%) hospital acquired infections [Hazard ratio: 0.427 (95% CI: 0.126–1.45) *P*-value: 0.1]. All cause mortality in the de-escalation group was 7.3% (*n* = 7) and in the non de-escalation group was 6% (*n* = 2) (Hazard ratio: 1.21, 95% CI: 0.273–5.41, *P*-Value 0.806). Out of seven deaths in the de-escalation group, three children had mortality attributed to sepsis and in the non de-escalation group, one child had mortality attributed to sepsis. (Hazard ratio: 1.04, 95% CI: 0.11–9.80, *P*-value: 0.96). Among children not eligible for de-escalation, 13% had death attributable to sepsis. Length of ICU stay in the de-escalation group was 4 days (IQR: 3–6.5 days) and in the non de-escalation group was also 4 days (IQR: 3–6 days) (*P*-value: 0.11). There were no significant differences in any of the outcomes between the two groups.

Commonest reason for de-escalation was the result of blood and body fluid cultures (62% of children with positive cultures and 57% of children with negative cultures underwent de-escalation. The other reasons for de-escalation were availability of alternate infective diagnosis like Dengue fever, respiratory viral infection, Scrub typhus and non-infective diagnoses like cardiac lesions, malignancies and immune related disorders (Table 3). Reluctance of the Consultant to de-escalate in patients with culture negative sepsis without any alternate diagnosis (81%) was the major factor for non de-escalation in the eligible group. The most common reason for reluctance was clinical improvement with empirical antibiotics. Delay in availability of culture sensitivity reports (18%) was another reason for non de-escalation within 72 h.

**Table 3:** Factors favoring antibiotic de-escalation.

**(*n* = 95)**	***n* (%)**
Microbiological confirmed infection	23 (24.2%)
Positive Real Time Polymerase Chain Reaction Testing	2 (2.1%)
Serology Testing for infections	7 (7.3%)
Non infective etiology	9 (9.4%)
Negative cultures and clinical improvement	54 (56%)
**Factors leading to non de-escalation (*****n*** **=** **33)**
Consultants' decision	27(81.8)%
Delay in availability of Culture reports	6 (18.2%)

## Discussion

De-escalation was possible in 59% of 162 episodes of sepsis in our study. Though there are no pediatric ICU studies to compare, the rate of de-escalation in our study is higher compared to other de-escalation studies. Hu-li and colleagues reported a de-escalation rate of 40% (15) in adult patients with VAP and a recent study by Mathieu et al. in patients with severe sepsis and septic shock has shown a de-escalation rate of 20% (16).

Culture yield was 22.9% in our study. Sigakis and colleagues' study on culture positive and negative sepsis has shown culture positivity of 11% among 10,393 septic patients (17). Studies have shown higher bacteriological yields in patients with septic shock and VAP (18, 19).

Clinical deterioration after antibiotic de-escalation was not different from the group with prolonged empirical antibiotic usage, suggesting the safety of de-escalation in our study. Very few de-escalation studies included clinical deterioration or clinical cure as outcome. Carugati et al. reported the clinical failure of antibiotics in community acquired pneumonia as 26.9% in de-escalated group and 40.9% in non de-escalated group (20). The DIANA study, a recent multicenter observational study, has shown more clinical cure with de-escalation (21).

Evidence was inconclusive regarding the occurrence of HAI after de-escalation (22–24). In our study, though 18% in the non de-escalated group developed HAI compared to 8% in de-escalated group, there was no statistically significant difference.

There is no significant difference in hospital mortality or sepsis attributed mortality among both the groups in our study. Two major meta-analyses were published in 2016 regarding de-escalation. Goh Ohji et al. analyzed 23 studies evaluating the effectiveness and safety of de-escalation therapy for a variety of infections. For critical outcomes such as in-hospital mortality, de-escalation appeared equally effective or even better than therapy that did not involve de-escalation (10). Paul et al. did a meta-analysis of 16 observational studies and three RCTs and found similar mortality between de-escalation and standard therapy groups. They found survival benefit with de-escalation in case of VAP (11). Silva et al. from Brazil in 2013 concluded that there is no adequate, direct evidence as to whether de-escalation of antimicrobial agents is effective and safe for adults with sepsis, severe sepsis or septic shock due to lack of good quality RCTs (25).

Antibiotic de-escalation, as already discussed has no consensual definition and there is no uniform protocol regarding the execution. There is no universal acceptance on the ranking of antibiotics by their spectrum of activity. Though cultures play a pivotal role in the de-escalation, there are multiple factors affecting the yield of cultures. Other than prior antibiotic usage, the yield of cultures depend on culturing practices like volume of blood, number of cultures, sterile way of taking culture and the standard of the microbiology lab. The clinician's motivation and knowledge toward antibiotic stewardship is crucial for standardizing antibiotic prescription and culturing practices in the ICU, which can result in sustainable de-escalation.

When we looked at the factors favoring de-escalation in our study, cultures turned out to have a major role, followed by other serological tests. Reluctance of the Consultant was the most common cause for not de-escalating followed by delayed availability of culture-sensitivity reports (Table 3). In Gonzalez and colleagues' study from France, appropriateness of the initial antibiotic therapy was the most common reason for de-escalation and multidrug resistance was the most common reason for non de-escalation (26). Salahudhin et al. found physicians were reluctant to de-escalate antibiotics in case of haemato-oncological patients, fungal or MDR infections and high baseline procalcitonin (27).

**Figure 2:**
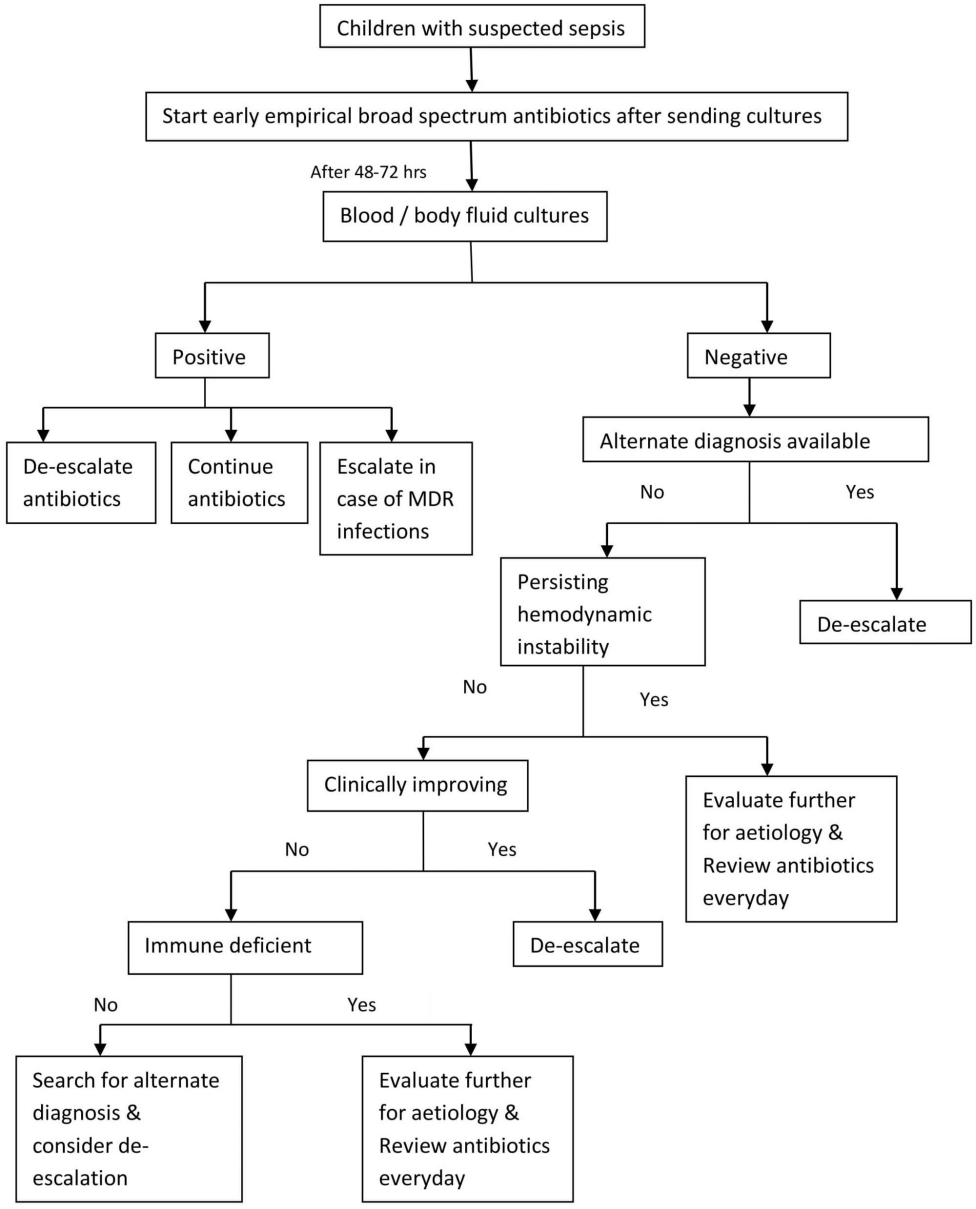
Suggested strategy for antibiotic de-escalation.

Multiple studies on de-escalation available in the literature have compared the de-escalated and non de-escalated groups without excluding the patients ineligible for de-escalation. De-escalating antibiotics in eligible patients and comparing this group with non-eligible cohort with higher severity of illness results in confounding bias favoring de-escalation group and may not be appropriate. Clinical cure or survival benefits conferred by less severity of the illness may get projected as benefits of de-escalation. In our study, to eliminate the effect of confounders, certain groups of children in whom de-escalation is outright unsafe were excluded from the analysis and we believe this is the strength of our study. This is in accordance with the patient flow pattern proposed by Silva and colleagues for de-escalation studies (25). In ICU, decisions like antibiotic de-escalation depends on factors like severity of illness, clinical improvement/ deterioration etc., which are very subjective. We have put all efforts to make the study protocol as objective as practically feasible in day to day ICU practice. We could not find studies addressing de-escalation in critically ill children and there are no guidelines available on de-escalation in culture negative sepsis. Strategic de-escalation framed based on the principles of de-escalation used in the study, represented as a flow chart for better understanding of de-escalation (Figure 2), is another strong and useful aspect of our study. The major limitations of our study are small numbers and the fact that it is a prospective observational study from a single center, rather than a RCT and it is not powered to detect the difference in outcome. Large size RCTs are required to better understand the effects of de-escalation.

## Conclusions

Early and appropriate antibiotic initiation is crucial for septic children. De-escalation of antibiotics based on microbiological and clinical evidence, appears to be a safe strategy to apply in critically ill children in intensive care units. However, large multicentric studies are needed to conclude on the safety of de-escalation. Motivation and knowledge about de-escalation gives confidence to clinicians and may result in increasing the rates of de-escalation.

## Data Availability Statement

The raw data supporting the conclusions of this article will be made available by the authors, without undue reservation.

## Ethics Statement

The studies involving human participants were reviewed and approved by Kanchi Kamakoti CHILDS Trust and Child Trust Medical Research Foundation Ethics Committee. Written informed consent from the participants' legal guardian/next of kin was not required to participate in this study in accordance with the national legislation and the institutional requirements.

## Author Contributions

VB: data collection, analysis, writing, reviewing and revising the manuscript. RK: conception of idea and reviewing the manuscript. PN: critical reviewing of the manuscript. BR: reviewing and revising the manuscript. All authors contributed to the article and approved the submitted version.

## Conflict of Interest

The authors declare that the research was conducted in the absence of any commercial or financial relationships that could be construed as a potential conflict of interest.

## Abbreviations

BL-BLI: Beta Lactam &#8211
Beta Lactamase Inhibitor
ICU: Intensive Care Unit
IQR: Inter Quartile Range
HAI: Hospital Acquired Infection
RCT: Randomized Control Trail
VAP: Ventilator Associated Pnuemonia.

## References

[1] Weiss SL Fitzgerald JC Pappachan J Wheeler D Jaramillo-Bustamante JC Salloo A. Global epidemiology of pediatric severe sepsis: the sepsis prevalence, outcomes, and therapies study. Am J Respir Crit Care Med. (2015) 191:1147–57. 10.1164/rccm.201412-2323OC 25734408 PMC4451622

[2] Kaur G Vinayak N Mittal K Kaushik JS Aamir M. Clinical outcome and predictors of mortality in children with sepsis, severe sepsis, and septic shock from Rohtak, Haryana: a prospective observational study. Indian J Crit Care Med. (2014) 18:437–41. 10.4103/0972-5229.136072 25097356 PMC4118509

[3] Ames SG Davis BS Angus DC Carcillo JA Kahn JM. Hospital variation in risk-adjusted pediatric sepsis mortality. Pediatr Crit Care Med. (2018) 19:390–6. 10.1097/PCC.0000000000001502 29461429 PMC5935525

[4] Weiss SL Peters MJ Alhazzani W Agus MSD Flori HR Inwald DP. Surviving sepsis campaign international guidelines for the management of septic shock and sepsis-associated organ dysfunction in children. Pediatr Crit Care Med. (2020) 21:e52–106. 10.1097/PCC.0000000000002198 32032273

[5] Centers for Disease Control and Prevention Office of Infectious Disease. Antibiotic Resistance Threats in the United States. (2013). Available online at: http://www.cdc.gov/drugresistance/threat-report-2013 (accessed January 28, 2015).

[6] Yang P Chen Y Jiang S Shen P Lu X Xiao Y. Association between antibiotic consumption and the rate of carbapenem-resistant gram-negative bacteria from China based on 153 tertiary hospitals data in 2014. Antimicrob Resist Infect Control. (2018) 7:137. 10.1186/s13756-018-0430-1 30479750 PMC6245771

[7] Ventola CL. The antibiotic resistance crisis: part 1: causes and threats. P T. (2015) 40:277–83. 25859123 PMC4378521

[8] Centers for Disease Control and Prevention. Antibiotic Stewardship Statement for Antibiotic Guidelines–Recommendations of the Healthcare Infection Control Practices Advisory Committee. (2016). Available online at: https://www.cdc.gov/hicpac/Pubs.Antibiotic-Stewardship-Statement.html (accessed April 05, 2017).

[9] Masterton RG. Antibiotic de-escalation. Crit Care Clin. (2011) 27:149–62. 10.1016/j.ccc.2010.09.009 21144991

[10] Ohji G Doi A Yamamoto S Iwata K. Is de-escalation of antimicrobials effective? A systematic review and meta-analysis. Int J Infect Dis. (2016) 49:71–9. 10.1016/j.ijid.2016.06.002 27292606

[11] Paul M Dickstein Y Raz-Pasteur A. Antibiotic de-escalation for bloodstream infections and pneumonia: systematic review and meta-analysis. Clin Microbiol Infect. (2016) 22:960–7. 10.1016/j.cmi.2016.05.023 27283148

[12] Harris PNA Tambyah PA Lye DC Mo Y Lee TH Yilmaz M. Effect of piperacillin-Tazobactam vs. Meropenem on 30-Day mortality for patients with e coli or klebsiella pneumoniae bloodstream infection and ceftriaxone resistance: a randomized clinical trial. JAMA. (2018) 320:984–94. 10.1001/jama.2018.12163 30208454 PMC6143100

[13] Goldstein B Giroir B Randolph A. International pediatric sepsis consensus conference: definitions for sepsis and organ dysfunction in pediatrics. Pediatr Crit Care Med. (2005) 6:2–8. 10.1097/01.PCC.0000149131.72248.E6 15636651

[14] Iroh Tam PY Bernstein E Ma X Ferrieri P. Blood culture in evaluation of pediatric community-acquired pneumonia: a systematic review and meta-analysis. Hosp Pediatr. (2015) 5:324–36. 10.1542/hpeds.2014-0138 26034164

[15] Li H Yang CH Huang LO Cui YH Xu D Wu CR. Antibiotics de-escalation in the treatment of ventilator-associated pneumonia in trauma patients: a retrospective study on propensity score matching method. Chin Med J. (2018) 131:1151–7. 10.4103/0366-6999.231529 29722334 PMC5956765

[16] Mathieu C Pastene B Cassir N Martin-Loeches I Leone M. Efficacy and safety of antimicrobial de-escalation as a clinical strategy. Expert Rev Anti Infect Ther. (2019) 17:79–88. 10.1080/14787210.2019.1561275 30570361

[17] Sigakis MJG Jewell E Maile MD Cinti SK Bateman BT Engoren M. Culture-negative and culture-positive sepsis: a comparison of characteristics and outcomes. Anesth Analg. (2019) 129:1300–9. 10.1213/ANE.0000000000004072 30829670 PMC7577261

[18] Kethireddy S Bilgili B Sees A Kirchner HL Ofoma UR Light RB. Culture-negative septic shock compared with culture-positive septic shock: a retrospective cohort study. Crit Care Me. (2018) 46:506–12. 10.1097/CCM.0000000000002924 29293143

[19] Kollef MH Ward S. The influence of mini-BAL cultures on patient outcomes: implications for the antibiotic management of ventilator-associated pneumonia. Chest. (1998) 113:412–20. 10.1378/chest.113.2.412 9498961

[20] Carugati M Franzetti F Wiemken T Kelley RR Kelly R Peyrani P. De-escalation therapy among bacteraemic patients with community-acquired pneumonia. Clin Microbiol Infect. (2015) 21:936.e11–8. 10.1016/j.cmi.2015.06.015 26115864

[21] De Bus L Depuydt P Steen J Dhaese S De Smet K Tabah A. Antimicrobial de-escalation in the critically ill patient and assessment of clinical cure: the DIANA study. Intensive Care Med. (2020) 46:1404–17. 10.1007/s00134-020-06111-5 32519003 PMC7334278

[22] Morel J Casoetto J Jospé R Aubert G Terrana R Dumont A. De-escalation as part of a global strategy of empiric antibiotherapy management. A retrospective study in a medico-surgical intensive care unit. Crit Care. (2010) 14:R225. 10.1186/cc9373 21167047 PMC3219998

[23] Eachempati SR Hydo LJ Shou J Barie PS. Does de-escalation of antibiotic therapy for ventilator-associated pneumonia affect the likelihood of recurrent pneumonia or mortality in critically ill surgical patients. J Trauma. (2009) 66:1343–8. 10.1097/TA.0b013e31819dca4e 19430237

[24] Leone M Bechis C Baumstarck K Lefrant JY Albanèse J Jaber S. De-escalation versus continuation of empirical antimicrobial treatment in severe sepsis: a multicenter non-blinded randomized noninferiority trial. Intensive Care Med. (2014) 40:1399–408. 10.1007/s00134-014-3411-8 25091790

[25] Silva BN Andriolo RB Atallah AN Salomão R. De-escalation of antimicrobial treatment for adults with sepsis, severe sepsis or septic shock. Cochrane Database Syst Rev. (2013) 2013:CD007934. 10.1002/14651858.CD007934.pub3 23543557 PMC6517189

[26] Gonzalez L Cravoisy A Barraud D Conrad M Nace L Lemarié J. Factors influencing the implementation of antibiotic de-escalation and impact of this strategy in critically ill patients. Crit Care. (2013) 17:R140. 10.1186/cc12819 23849321 PMC4055984

[27] Salahuddin N Amer L Joseph M El Hazmi A Hawa H Maghrabi K. Determinants of deescalation failure in critically ill patients with sepsis: a prospective cohort study. Crit Care Res Pract. (2016) 2016:6794861. 10.1155/2016/6794861 27493799 PMC4963586

